# Association of gestational diabetes mellitus with changes in gut microbiota composition at the species level

**DOI:** 10.1186/s12866-021-02207-0

**Published:** 2021-05-14

**Authors:** Fang Chen, Yu Gan, Yingtao Li, Wenzhi He, Weizhen Wu, Kejian Wang, Qing Li

**Affiliations:** Department of Obstetrics and Gynecology, Key Laboratory for Major Obstetric Diseases of Guangdong Province, The Third Affiliated Hospital of Guangzhou Medical University, Guangzhou, China

**Keywords:** Gestational diabetes mellitus, Gut microbiota, Bacterial species, 16S rRNA microarray

## Abstract

**Background:**

Gestational diabetes mellitus (GDM), a common endocrine disorder with rising prevalence in pregnancy, has been reported to be associated with alteration of gut microbiota in recent years. However, the role of gut microbiome in GDM physiopathology remains unclear. This pilot study aims to characterize the alteration of gut microbiota in GDM on species-level resolution and evaluate the relationship with occurrence of GDM.

**Methods:**

An analysis based on 16S rRNA microarray was performed on fecal samples obtained from 30 women with GDM and 28 healthy pregnant women.

**Results:**

We found 54 and 141 differentially abundant taxa between GDM and control group at the genus and the species level respectively. Among GDM patients, *Peptostreptococcus anaerobius* was inversely correlated with fasting glucose while certain species (e.g., *Aureimonas altamirensis*, *Kosakonia cowanii*) were positively correlated with fasting glucose.

**Conclusions:**

This study suggests that there are large amounts of differentially abundant taxa between GDM and control group at the genus and the species level. Some of these taxa were correlated with blood glucose level and might be used as biomarkers for diagnoses and therapeutic targets for probiotics or synbiotics.

**Supplementary Information:**

The online version contains supplementary material available at 10.1186/s12866-021-02207-0.

## Introduction

Gestational diabetes mellitus (GDM) is defined as a glucose intolerance resulting in hyperglycemia of variable severity during pregnancy [1, 2]. It is associated with short-term obstetric and perinatal complications (e.g., preeclampsia, increased cesarean delivery rates, macrosomia, birth injury) and considered to increase the long-term health risks (e.g., cardiovascular, obesity and type 2 diabetes) for the mother and the offspring [3, 4]. Due to changes in hormones such as progesterone, estrogen and placental factors, insulin sensitivity naturally declines with advancing gestation during pregnancy. In this situation, a compensatory increase in insulin secretion usually maintains a normal glucose homeostasis. However, GDM occurs if pancreatic β-cells fail to meet the demand of insulin secretion [2].

GDM affects nearly 16.5% of pregnancies worldwide, and this number tends to increase as the obesity epidemic escalates [3]. The Hyperglycemia and Adverse Pregnancy Outcome (HAPO) study involving 15 multinational centers reported a prevalence of GDM between 9.3 and 25.5% in the global population [5]. The GDM incidence in China is also alarming, with a recent systematic review implicated a pooled GDM prevalence of 14.8% in the Chinese population [6].

Intestinal bacteria have been suggested to play important roles in the host metabolism, especially in diabetes [7–9]. Thus, it is necessary to further explore the relationship between gut microbiota and GDM. Differential abundant taxa might be used as biomarkers for early diagnosis or targets for probiotic intervention. A number of studies have reported that the significant alterations of gut microbiota in women with GDM were associated with abnormal glucose metabolism [10–13]. Nonetheless, the link between gut microbiome and GDM is still ambiguous and needs to be clarified [14]. So far, only one study used the shotgun metagenomics sequencing method to analyze the microbial species in GDM [10], other published results were based on 16S rRNA sequencing technique that only covered taxa at genus level or above [15]. Given the limitation of most previous research on gut microbiota of GDM, we applied innovative microarray technique rather than sequencing platform for its prominent microbial species level resolution, which helped to better explain the gut dysbiosis [16, 17].

## Method

### Materials and methods

#### Study design and sample collection

A cohort study was conducted to analyze the role of intestinal microbiota in the development of GDM. The protocols of this study were approved by the Ethics Committee of The Third Affiliated Hospital of Guangzhou Medical University (GD2019–033). Informed, written consent was given by all volunteers in accordance with the protocol. In addition, all methods were carried out in accordance with the relevant guidelines and regulations. In our study, the pregnant women of 22–45 years of age were enrolled. Women who had the following criteria were excluded from our study: taking probiotics, use of antibiotics or other drugs within 1 month, complications of delivery pregnancy-induced hypertension, intestinal diseases, acute gastroenteritis, autoimmune, thyroid dysfunction, liver and kidney disease. Eventually, the study cohort consisted of 58 participants including 30 women with GDM and 28 health gravida, one of whom was twin pregnancy, the rest were singleton pregnancy.

The 2-h 75 g oral glucose tolerance test (OGTT) was examined in women in their third trimester (24–28 gestation weeks) according to the International Association of the Diabetes and Pregnancy Study Groups (IADPSG) criteria for GDM [18, 19]. The participants were required to eat no less than 250 g carbohydrates 3 days before the OGTT test and at least 8 h-overnight fast before collecting the venous blood. Anthropometric measurements, basic information and the family history were obtained at the same day. Pregnant women were diagnosed with GDM if one or more of the following OGTT result were met: fasting plasma glucose (FPG), ≥5.1 mmol/liter; 1 h glucose, ≥10.0 mmol/liter; 2 h glucose, ≥8.5 mmol/liter [20]. Overweight was defined as body mass index (BMI) ≥ 25.0 kg/m^2^ and obese was defined as BMI ≥ 28.0 kg/m^2^ [21]. After instructed by pre-recorded video, the participants were required to collect at least 500 mg stool samples into sterilized sample tubes with preservative solution (Halgen, China) within 24–48 h before OGTT or dietary adjustment after diagnosis of GDM. Bacterial DNA was extracted using Halgen Stool Isolation kit (Halgen, China) according to the manufacturer’s protocol. Then DNA amplification and array hybridization were performed following the methods detailed in the previous study [16]. Venous blood was collected in the fasting state for metabolic biomarkers. Plasma glucose was analyzed by Cobas 8000 modular analyzer (Roche Diagnostics Ltd., Rotkreuz, Switzerland). Body weight and high was verified by the pregnant women’s self-report. Pregnancy trimester were determined by first semester ultrasound and last menstrual period.

#### Bacterial DNA extraction and microarray hybridization

Bacterial DNA was extracted from the stool samples by using the Stool DNA Extraction Kit (Halgen, China) according to the product instruction manual. DNA amplification and labeling were carried out following the previously published methods [16] before array hybridization experiments. According to our previous study [16], DNA products were hybridized with probes of the microarray (Halgen, China) designed for the entire variable regions of bacterial 16S rRNA. The relative abundance of each bacterial species was measured by the mean of the Cy5/Cy3 ratios of the corresponding species-specific probes.

#### Data analysis

The clinical features were analyzed using Pearson chi-square test or Fisher’s exact test as appropriate for categorical variables and independent Student’s t-test or Rank sum test was used as appropriate for continuous variables. Correlations were calculated using Spearman’s rank correlations. The microarray data was produced by the specialize program transforming the hybridization signal to relative abundance value following the methods described in our previous publication [16]. The α-diversity was calculated using and QIIME software [22] with default parameters and further compared by Wilcoxon rank-sum test. PCoA and NMDS analyses were performed by QIIME modules and visualized by R packages (version 3.5.2). Linear Discriminant Analysis (LDA) Effect Size (LEfSe) [23] analysis was performed to identify taxonomic biomarkers that characterize the differences between pregnant women with and without GDM. The *p*-value for each species were calculated by Wilcoxon test.

## Result

### Characteristics of subject recruitment

A total of 58 stool samples were collected from 30 women with GDM and 28 normoglycemic female control subjects. As shown in Table 1, there are no significant difference between two groups in demographic characteristics including height, weight, BMI before pregnancy, gestational weight gain, and the gestational week at examination. As expected, markers of glucose tolerance such as fasting plasma glucose (FPG), 75 g OGTT 1-h postprandial glucose (1 h-PG) and 2-h postprandial glucose (2 h-PG) were higher in the GDM group than in the control group (Student’s t-test *P* value: 0.011, 0.001, 0.001, respectively).

**Table 1:** Clinical characteristic and OGTT results of GDM and control groups

Characteristic	Women with GDM (*n* = 30)	Normoglycemic women (*n* = 28)	*P* value
Age (year)	32.80 (5.22)	30.29 (4.50)	0.055
Ht (cm)	159.43 (5.47)	157.43 (3.89)	0.112
Wt (kg) pregnancy	57.78 (10.06)	53.62 (9.43)	0.098
BMI (kg/m^2^) pregnancy	22.68 (3.45)	21.61 (3.53)	0.173
Overweight or Obese (BMI ≥ 28 kg/m^2^) pregnancy	3 (10.00%)	1 (3.60%)	0.650
Gestational weight gain (kg) at OGTT	42.44 (7.24)	38.98 (6.05)	0.061
Fasting glucose (mmol/liter)	4.56 (0.48)	4.28 (0.33)	0.011*
1 h-PG (mmol/liter)	10.32 (0.81)	7.32 (1.27)	0.001*
2 h-PG (mmol/liter)	8.74 (1.32)	6.45 (0.65)	0.001*
Gestational wk. (kg) at examination	25.4 (1.01)	25.22 (1.58)	0.599

The *P* value calculated by Chi-Squared test or t test. “*” mean *p* < 0.05. Overweight or Obese rate (BMI ≥ 28 kg/m^2^) pregnancy was shown as n (%), other results were shown as mean (SD)

### Overall differences between GDM and control group

**Fig. 1:**
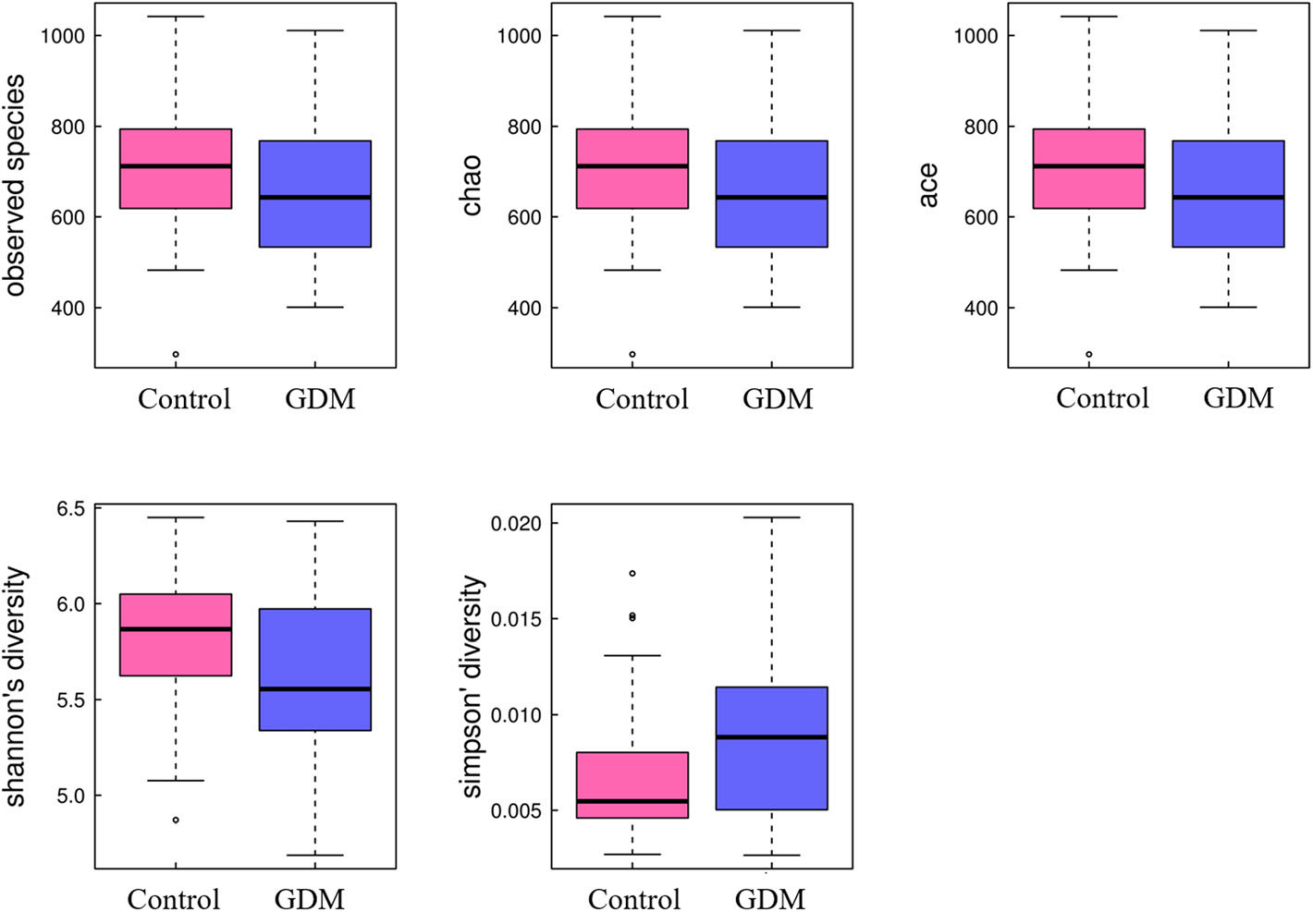
The α-diversity of gut microbiome in GDM and control groups. The α-diversity of GDM group were lower albeit not statistically significance, Wilcoxon rank-sum test *P* value for Observed species, Chao, Ace, Shannon and Simpson are 0.144, 0.144, 0.144, 0.075, 0.067, respectively

**Fig. 2:**
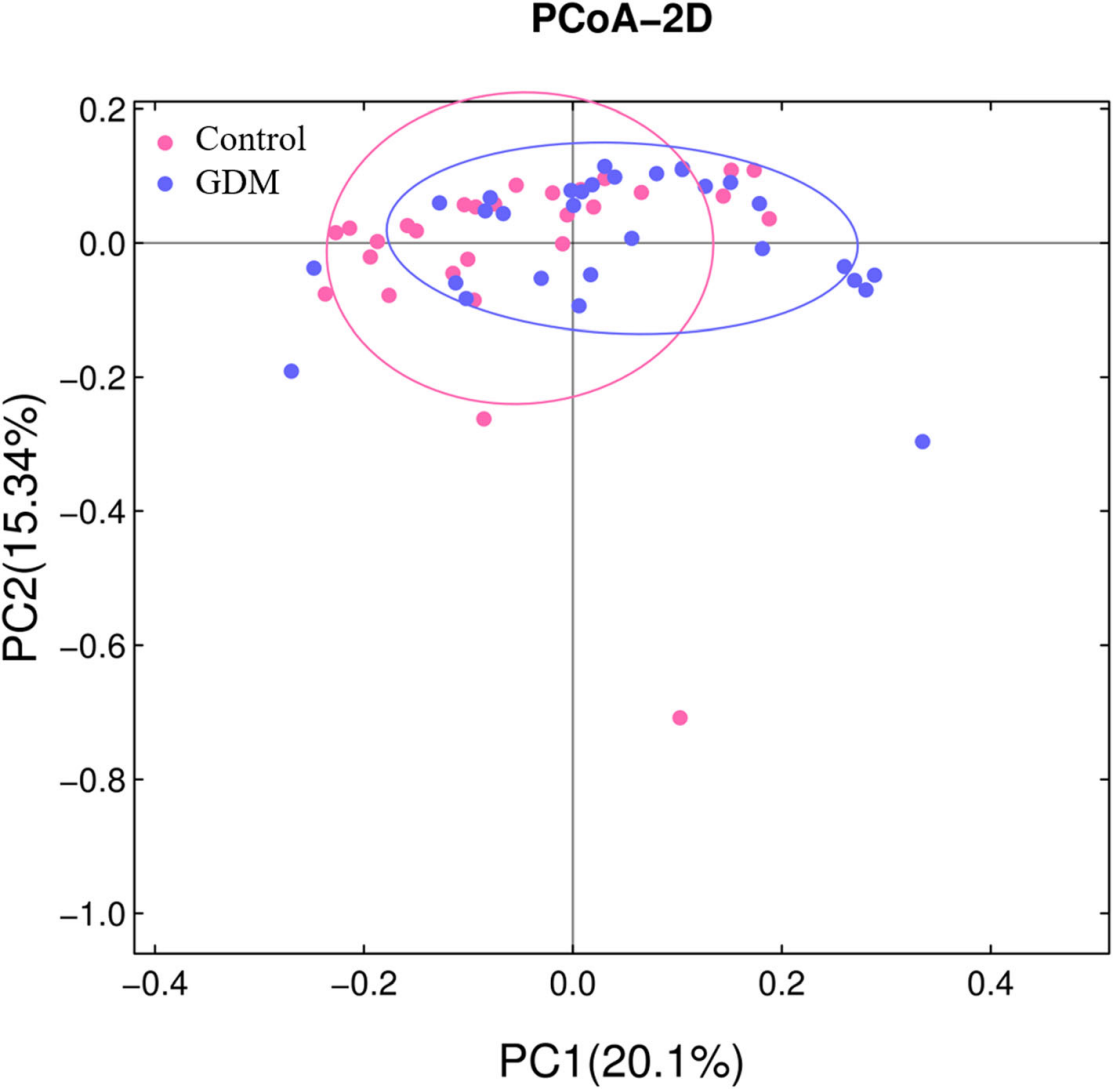
Bray Curtis Principal Coordinate (PCoA) analysis for gut microbiome of GDM and control groups. The two components of Bray Curtis PCoA plot explained 20.1 and 15.34% of the variants. ADONIS statistic: *R*^*2*^ = 0.03384, *P* = 0.017

A total of 1234 unique OTUs were identified in all samples, including 1117 OTUs shared by both groups. We found that the α-diversity of GDM group were slightly but not significantly lower than that of control group (Fig. 1). On the other hand, Bray-Curtis distance-based analysis of β-diversity showed a partial separation of GDM and control groups, indicating that the microbiota structure differed between GDM and normoglycemic women (Fig. 2, ADONIS statistic: *R*^*2*^ = 0.03384, *P* value =0.017).

### Bacterial species with differential abundance

**Fig. 3:**
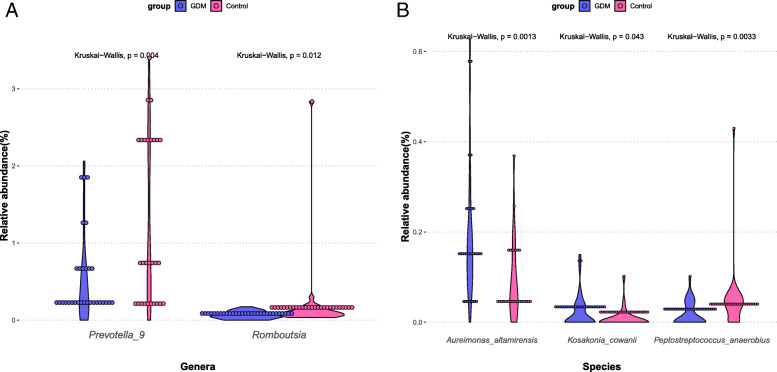
The relative abundance of part of differentially abundant taxa in GDM and control groups. **a** Part of the differentially abundant genera between GDM and control groups. **b** Part of the differentially abundant species between GDM and control groups

At the genus level, we found 54 genera with differential abundance between GDM and control group (Additional file 1), including 42 genera depleted in GDM (e.g., *Prevotella* and *Romboutsia*) (Fig. 3a). At the species level, there were 37 species significantly enriched in GDM patients such as, *Corynebacterium* spp. (*Corynebacterium appendicis, Corynebacterium coyleae, Corynebacterium durum, Corynebacterium frankenforstense, Corynebacterium freneyi, Corynebacterium glaucum, Corynebacterium kroppenstedtii, Corynebacterium xerosis*), *Lactobacillus* spp. (*Lactobacillus ceti, Lactobacillus sanfranciscensis, Lactobacillus vaccinostercus*) and *Blautia hydrogenotrophica*. Meanwhile, we also found 104 species enriched in normoglycemic pregnant women such as *Bacteroides* spp. (*Bacteroides acidifaciens, Bacteroides intestinalis, Bacteroides nordii, Bacteroides plebeius, Bacteroides salyersiae*), *Bacillus* spp. (*Bacillus anthracis, Bacillus clausii, Bacillus idriensis, Bacillus massilioanorexius*), *Bifidobacterium* spp. (*Bifidobacterium bifidum, Bifidobacterium gallicum, Bifidobacterium longum*), *Clostridium* spp. (*Clostridium pasteurianum*, Clostridium saccharogumia), *Eubacterium* spp. (*Eubacterium hallii, Eubacterium multiforme*), *Prevotella* spp. (*Prevotella falsenii, Prevotella maculosa, Prevotella nigrescens, Prevotea oris, Prevotella paludivivens, Prevotella stercorea*) (Fig. 3b). More details can be found in Additional file 2.

### Species correlated with glucose tolerance in GDM

**Fig. 4:**
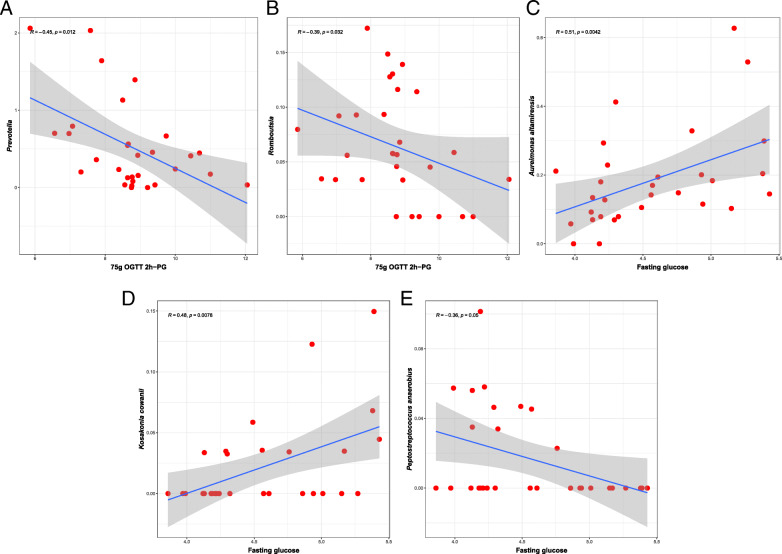
The caption was rephrased as follow: Fig. 4. Correlation between part of differential abundant taxa with glucose tolerance indicators. **a** *Prevotella* and **b** *Romboutsia* genus was negatively correlated with 75 g OGTT 2 h-PG in women with GDM. **c** *Aureimonas altamirensis* and **d** *Kosakonia cowanii* was positively correlated with fasting glucose level in women with GDM. **e** *Peptostreptococcus anaerobius* was negatively correlated with fasting glucose level in women with GDM

To further understand the relationship between gut microbiome and GDM, we evaluated the correlations between the differentially abundant taxa and clinical traits. At genus level, *Prevotella* and *Romboutsia* were negatively correlated with 2 h-PG in75 g OGTT in GDM group. At species level, we found some species positively correlated with fasting glucose in GDM patients, such as *Aureimonas altamirensis, Kosakonia cowanii*. On the other hand, *Peptostreptococcus anaerobius* was negatively correlated with fasting glucose in GDM group (Fig. 4, Additional file 3). These correlations were consistent with the taxonomy abundance difference between two groups and implied the potential critical role in GDM pathophysiology.

## Discussion

The correlation between alterations of gut microbiome and GDM has been repeatedly reported [10–13, 24]. However, there is little knowledge about the composition of gut microbiota in GDM at the species level. By comparing 30 GDM patients and 28 normoglycemic pregnancy, we found the alterations in gut bacterial species and further explored their association with glucose intolerance. Our results provided more specified insights into the pathology of GDM.

By analyzing the differential bacterial species between two groups, we observed the beneficial acetate and lactate-producing bacteria (e.g., *Bifidobacterium* spp.) and butyrate-producing bacteria (e.g., *Eubacterium* spp.) depleted in GDM patients. These results were in line with previous in-vitro studies suggesting that short-chain fatty acids (SCFAs) may be able to improve insulin sensitivity and prevent inflammation induced by sterile or bacterial inflammation [25, 26]. Moreover, we found that *Blautia hydrogenotrophica* was more abundant in women with GDM. *Blautia* was reported to be the dominant genus with enriched abundance in glucose-intolerant individuals [27] and associate with high BMI [28]. Our finding was consistent with previous study reporting the elevated abundance of *Blautia* in GDM patients [4].

Previous studies showed that elevated *Prevotella* genus may contribute to impair gut permeability in women with GDM by increasing mucin oligosaccharide degradation [28–30]. In contrast, our results suggested the depletion of *Prevotella* genus and some *Prevotella spp*. in GDM. The *Prevotella* genus was found to be highly prevalent in non-Westerners who consume a plant-rich diet [31, 32]. Moreover, our results showed the negative association between the *Prevotella* genus and glucose intolerance indicator such as 2 h-PG in 75 g OGTT. It has been shown that *Prevotella* genus can improve glucose metabolism stimulated by the intake of prebiotics [33]. Thus, we speculated that the enriched *Prevotella* spp. including *Prevotella falsenii, Prevotella maculosa, Prevotella nigrescens, Prevotella oris, Prevotella paludivivens* and *Prevotella stercorea* may contribute to the higher fiber-rich diet consumption and indicates their beneficial roles in glucose metabolism. However, a minority of *Prevotella* spp., such as *Prevotella aurantiaca* was enriched in GDM, this may be explained by the high species and function diversity of *Prevotella* genus [34]. In general, these results shed more light on the ambiguous role of *Prevotella* genus within the intestinal microbiota and their effects on the host. Likewise, we also found that several *Bacteroides* spp. were enriched in GDM women but the majority of *Bacteroides* genus are enriched in control group. Similar situation also existed in other genera in our study. Thus, the association of gut microbiome at the genus level may cause oversimplified vision that does not take into account sub-genus diversity.

Additionally, our results showed that the reduced *Romboutsia* genus was associated with higher 2 h-PG in 75 g OGTT in GDM patients. Mangifesta et al. reported that *Romboutsia* genus was more abundant in healthy mucosa samples compared with that from polyps-associated or colorectal cancer tissue, which indicated that the depletion of this genus was associated with disease condition [35]. We assumed that decreased *Romboutsia* genus may be involved in the occurrence of GDM via changing the gastrointestinal mucosa permeability.

Furthermore, some species were positively correlated with fasting glucose in GDM patients, such as *Aureimonas altamirensis, Kosakonia cowanii*. These species were reported to be potential pathogens of infection, as a risk factor for causing GDM via chronic low-grade inflammation [36–39]. Whereas *Peptostreptococcus anaerobius* was negatively correlated with fasting glucose in GDM group. Previous findings provided supportive evidence that the abundance of *P. anaerobius* was significantly increased in type 2 diabetes after weight loss intervention compared with lean and obese controls [40]. Although *P. anaerobius* was considered to be widely distributed in human gut microbiota contributing to systemic infection [40], our results implied that *P. anaerobius* might play a crucial role in balancing glucose metabolism. These findings need further investigation to determine the role of above discussed taxonomy in GDM.

Applying the innovative microarray technique rather than the sequencing platform, we found large amounts of differentially abundant taxa between GDM and control group at the species level resolution, which broadens our understanding of gut dysbiosis in GDM women. However, some limitations of this study need to be addressed. First, due to the small sample size and single-centered nature, external validation is recommended for future study. Second, we cannot entirely exclude the influence of potential confounders such as diet and gravidity history on gut microbiota. Well-controlled clinical studies addressing potential confounders will be needed to validate our findings.

## Conclusion

This study suggests that there are large amounts of differentially abundant taxa between GDM and control group at the genus and the species level. Some of these taxa were correlated with blood glucose level and might be used as biomarkers for diagnoses and therapeutic targets for probiotics or synbiotics.

## Supplementary Information

**Additional file 1.** The differential taxonomy at genus level between the two groups, Wilcoxon test *P* value < 0.05.

**Additional file 2.** The differential taxonomy at species level between the two groups, Wilcoxon test *P* value < 0.05.

**Additional file 3.** Differential abundant Genera and Species correlated with glucose tolerance indicators in GDM patients, Spearman’s rank correlations < 0.05.

## Data Availability

The datasets used and/or analysed during the current study are available from the corresponding author on reasonable request.

## Abbreviations

GDM: Gestational diabetes mellitus
OGTT: Oral glucose tolerance test
SCFAs: Short-chain fatty acids
FPG: Fasting plasma glucose
1 h-PG: 1-h postprandial glucose
2 h-PG: 2-h postprandial glucose
