# Supplementary material for: Association of gestational diabetes mellitus with changes in gut microbiota composition at the species level
Source: BMC Microbiol. 2021 May 14;21:147. doi: 10.1186/s12866-021-02207-0 (PMC8122539; doi:10.1186/s12866-021-02207-0)
Supplement: Supplementary file 3 — Additional file 3. Differential abundant Genera and Species correlated with glucose tolerance indicators in GDM patients, Spearman’s rank correlations < 0.05. [file 12866_2021_2207_MOESM3_ESM.docx]

Supplementary Table 3. Differential abundant Genera and Species correlated with glucose tolerance indicators in GDM patients

|  | Fasting glucose | Spearman coefficient rho | P value | OGTT at 1h | Spearman coefficient rho | P value | OGTT at 2h | Spearman coefficient rho | P value |
| --- | --- | --- | --- | --- | --- | --- | --- | --- | --- |
| Genus | *Aureimonas* | 0.508 | 0.004 | - | - | - | *Prevotella_9* | -0.451 | 0.012 |
|  | *Kosakonia* | 0.476 | 0.008 | - | - | - | *Romboutsia* | -0.392 | 0.032 |
| Species | *Aureimonas altamirensis* | 0.508 | 0.004 | *Bacteroides nordii* | 0.373 | 0.042 | *Clostridium dakarense* | -0.404 | 0.027 |
|  | *Kosakonia cowanii* | 0.476 | 0.008 | *Corynebacterium coyleae* | -0.435 | 0.016 | - | - | - |
|  | *Peptostreptococcus anaerobius* | -0.362 | 0.049 | *Lactobacillus herbarum* | 0.373 | 0.042 | - | - | - |
|  | *-* | - | - | *Methylobacterium populi* | -0.555 | 0.001 | - | - | - |
